# Surgical technique of robotic arm-assisted total knee arthroplasty via the lateral parapatellar approach for valgus knee deformity

**DOI:** 10.1186/s42836-026-00387-6

**Published:** 2026-05-05

**Authors:** Yasushi Oshima, Hiroki Yoshida, Tokifumi Majima

**Affiliations:** Department of Orthopaedic Surgery, Nippon Medical School, 1-1-5 Sendagi, Bunkyo-Ku, Tokyo, 113-8603 Japan

**Keywords:** Valgus knee deformity, Total knee arthroplasty, Robotic technology, Lateral parapatellar approach, Onlay oval patellar implant, Patellar tracking

## Abstract

**Background:**

Valgus knee deformity, which is less common, is not a mirror image of varus knee deformity and poses unique technical challenges in total knee arthroplasty (TKA). Although the lateral parapatellar approach may be advantageous for severe valgus knee, the medial approach is often preferred due to surgeons’ limited familiarity with valgus TKA and the lateral approach. Recently, robotic technology has demonstrated superior accuracy in bone resection and soft-tissue balancing during TKA. Hence, we introduce the application of robotic technology for valgus knees via the lateral approach in TKA. As the standard patella drill template of the onlay oval patellar implant was designed for the medial approach, we created a reversed-asymmetric patella drill template for the lateral approach. In the recent cases, patellar tracking following prosthesis implantation was also evaluated using robotic technology.

**Methods:**

We included cases of primary TKA performed for Ranawat classification types II and III with uncorrectable valgus knee alignment, as well as for valgus deformity > 20°. In TKA, arthrotomy was performed via the lateral approach, and the patella was retracted medially. After soft-tissue balancing was adjusted, bone resection was performed using the Mako robotic system. The patella was replaced with an onlay oval patellar implant using our novel patella drill template in the lateral approach. Patellar tracking on the femoral trochlear groove after implantation was visualized and assessed using robotic technology.

**Results:**

The surgical procedures were performed smoothly in 10 knees of 9 patients. The pre-operative limitations of knee extension, Visual Analog Scale scores, and radiographic knee alignment significantly improved following TKA. Pre-operatively, the tibiofemoral joint gaps were tighter laterally in both extension and flexion; post-operative medial laxity was effectively corrected. The accuracy and precision of prostheses positioning were confirmed radiographically. Patellar tracking was found to be appropriate after replacement with the oval patellar implant.

**Conclusions:**

The combination of robotic assistance, the lateral approach, and onlay oval patellar implants using our originally developed patella drill template showed feasibility for precise bone resection, optimal soft-tissue balancing, and proper patellar tracking for TKA in cases of valgus knee deformity.

## Background

The number of total knee arthroplasty (TKA) procedures performed for advanced or end-stage knee osteoarthritis (OA) in older patients has increased substantially worldwide due to the aging of the population [1, 2]. Most cases involve medial compartment OA presenting as varus knee deformity, whereas TKA for lateral compartment OA with valgus knee deformity accounts for only approximately 10%–15% of all cases [3, 4].

In varus knees, the medial soft-tissue structures become pathologically tight, whereas the lateral soft tissues are relatively lax, further accentuating the imbalance of the knee joint. To achieve proper lower-extremity alignment and optimal soft-tissue balancing during TKA for varus knees, a step-by-step, selective release technique of the medial structures has been developed. Traditionally, this approach aims to create a rectangular and symmetrical tibiofemoral gap, which is considered essential for stable joint kinematics [5, 6].

In valgus knees, the medial soft tissues tend to be elongated, whereas the lateral soft tissues (including the iliotibial band [ITB], lateral collateral ligament, and lateral retinaculum) are contracted, often resulting in medial instability. Additionally, valgus knees are characterized by lateral tibiofemoral compartment bone loss, hypoplasia of the lateral femoral condyle, and femoral rotational deformity, all of which contribute to patella maltracking compared with varus knees [7]. Because the anatomical and soft-tissue characteristics of valgus knees differ fundamentally from those of varus knees, TKA for valgus deformity cannot simply be approached as a mirror image of varus TKA and remains a technically demanding procedure [8–10].

The standard surgical approach for varus knees is the medial parapatellar approach; however, in valgus knees, both medial and lateral parapatellar approaches can be employed [11]. The lateral approach offers direct visualization of the lateral compartment deformity and facilitates more precise adjustment of the contracted lateral soft tissues [4, 9]. However, because of the relative rarity of valgus knee TKA and limited familiarity among surgeons, this approach is often avoided. In Japan, for example, the lateral approach has been reported to be used in only 0.5% of primary TKA cases, compared with 70.7% for the medial parapatellar approach [12].

Robotic technology has rapidly evolved in recent years and has gained considerable attention for its ability to enhance surgical accuracy while requiring a relatively short learning curve in TKA [13]. The adoption of robotic arm-assisted TKA (RTKA) has increased considerably over the past decade, and by 2023, the number of RTKA procedures in Australia surpassed those of both conventional manual TKAs and computer navigation-assisted TKAs [14]. We hypothesized that the use of robotic technology could enable accurate and reproducible outcomes even in cases of valgus knee deformity managed through the lateral approach.

Regarding patellar resurfacing, several implant designs have been developed in recent years, including inset and onlay types, as well as round (symmetric) and oval (asymmetric) shapes, to better match the contour of the femoral trochlea and facilitate more natural patellar tracking. Although no consensus has been reached on the optimal design, onlay oval implants have been shown to provide smaller lateral tilt angles, reduced rates of lateral patellar contact, superior bone coverage, lower rates of lateral facetectomy, and decreased lateral underhang compared with other designs [15]. Consequently, onlay oval implants are considered to offer improved biomechanical compatibility and may reduce anterior knee pain after TKA [16]. In the medial approach, patella resurfacing is typically performed by everting the patella laterally and positioning the patella drill template from the medial side. However, in the lateral approach, these directions are reversed, rendering the existing standard patella drill template for onlay oval implants unusable. To resolve this issue, we designed and implemented an original reversed-asymmetric patella drill template specifically for use with the oval implant for the lateral approach.

Traditionally, the no-thumb test has been used to assess patellar tilt; however, this method makes it difficult to accurately evaluate patellar tracking along the femoral trochlear groove [17]. Therefore, in this study, we utilized robotic technology to directly visualize patellar tracking on a computer monitor.

Here, we describe the application of robotic technology for valgus knees via the lateral approach in TKA. We also introduce a novel patella drill template for onlay oval patellar implants in the lateral approach and demonstrate assessment of patellar tracking using robotic technology.

## Methods

### Surgical indication

Moderate (grade III) and severe (grade IV) knee OA patients with Kellgren–Lawrence (KL) classification [18] whose condition was resistant to conservative treatments were indicated for primary TKA using the Mako robotic system (Stryker, Mahwah, NJ, USA). In patients with valgus knee deformity, the severity was assessed according to the Ranawat classification [8]. In this system, type I represents a minimal deformity (< 10° valgus) with correctable alignment on stress testing and an intact, functional medial collateral ligament (MCL). Type II denotes a more pronounced deformity (10°–20° valgus) in which the MCL is functionally elongated but retains a firm endpoint; this category may also include cases with minimal deformity but uncorrectable alignment. Type III indicates a severe osseous deformity (> 20° valgus) with an absent or markedly attenuated MCL. In our institution, the medial approach is generally used for type I deformities, whereas the lateral approach is employed for types II and III. Additionally, constrained or hinged prostheses are prepared for types II and III, and are implanted if knee instability persists.

**Fig. 1:**
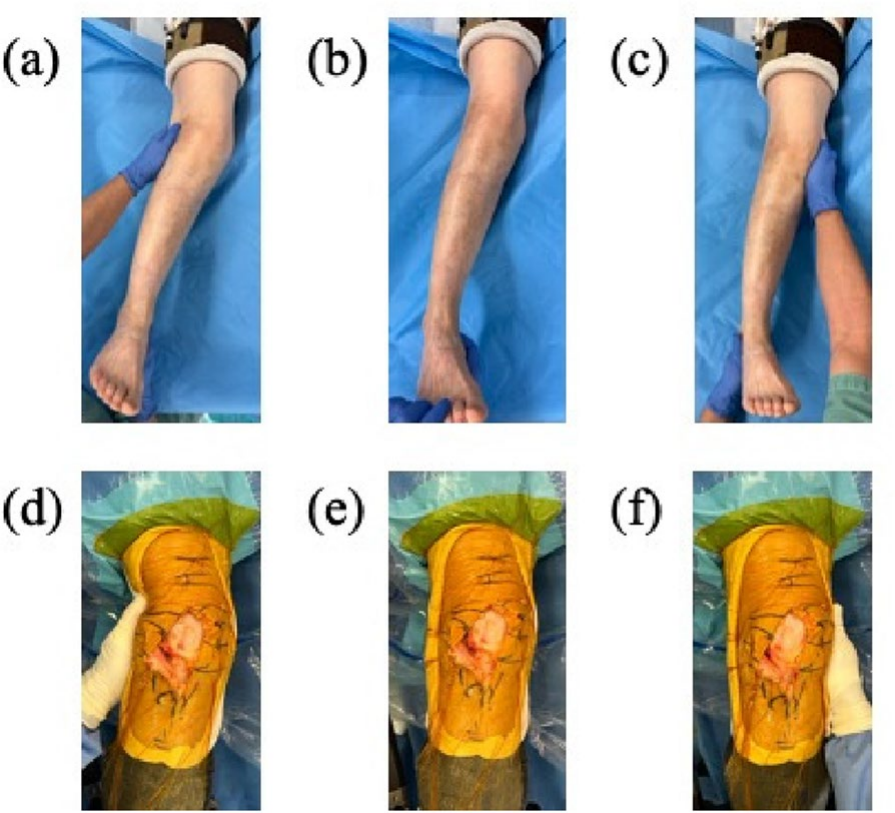
Manual stress test. (**a**) valgus stress test, (**b**) neutral position, and (**c**) varus stress test under general anesthesia. Valgus knee alignment remained under the varus stress test. (**d**) Valgus stress test, (**e**) neutral position, and (**f**) varus stress test after the lateral approach. The remaining valgus deformity with the varus stress test was diminished

The manual varus-valgus stress test was performed before surgery under general anesthesia. Uncorrectable valgus alignment was diagnosed when the valgus deformity remained during the manual varus stress test (Fig. 1a–c). Subsequently, the indication for the lateral approach was determined.

### Surgical procedures

All TKAs were performed by a single surgeon at a single institution under general anesthesia using an air tourniquet. After an approximately 12 cm midline longitudinal skin incision was extended from two finger-breadths proximal to the superior border of the patella to the tibial tuberosity, knee arthrotomy was performed via the lateral approach. At this time, the remaining valgus deformity with the varus stress test decreased (Fig. 1d–f). Distal femoral and proximal tibial osteotomies were conducted using a mechanical alignment technique. Cruciate-retaining (CR) or posterior-stabilized (PS) implants (Triathlon, Stryker, Mahwah, NJ, USA) were selected based on the pre-operative knee range of motion (ROM), severity of knee malalignment, and condition of the posterior cruciate ligament. The patella was resurfaced in all PS cases and in CR cases with moderate to severe patellar deformity. All components were fixed with bone cement.

### Robotic technology in TKA

#### Pre-operative RTKA preparation

A computed tomography (CT)-based, surgeon-controlled, semi-active robotic system with visual, tactile, and auditory feedback (Mako, Stryker) was used. CT images of the full length of the affected lower extremity were obtained using the standard Mako protocol. Next, pre-operative three-dimensional surgical planning was created and uploaded into the Mako system.

#### Intra-operative RTKA preparation

**Fig. 2:**
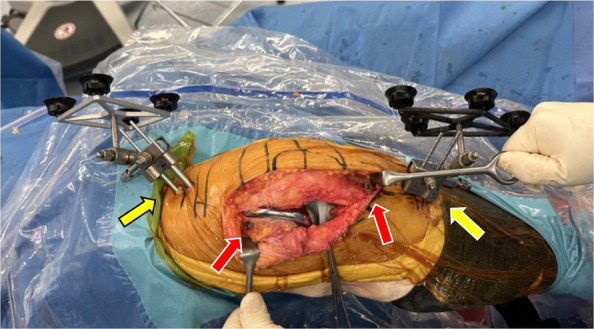
Preparation of the Mako system. Two bone pins with a reference array (yellow arrows) and one checkpoint pin (red arrows) were inserted into the femur and tibia

Two bicortical bone pins (3.2 mm in diameter) were inserted in the distal femur and proximal tibia, respectively, and reference arrays were mounted to enable dynamic tracking of the knee. Distal femoral and proximal tibial checkpoint pins (2.2 mm in diameter) were inserted hemicortically to verify registration accuracy; the femoral pin was placed in the lateral femoral cortex (Fig. 2). In cases with anticipated metal augmentation and stem extension for lateral bone defects, registration and checkpoint pins were positioned sufficiently away from the joint line to avoid interference with the implants [19]. Bone registration was completed by mapping anatomical landmarks displayed on the computer monitor, thereby confirming alignment and establishing bone geometry for the Mako system.

After the removal of femoral and tibial osteophytes and the cruciate ligament(s), medial and lateral tibiofemoral joint gaps were measured in near-extension (10° flexion from the maximal extension) and 90° flexion under manual varus and valgus stress tests in extension and using dedicated gap spoons in flexion, respectively, following the standard Mako surgical procedure. Medial soft tissues are usually stretched in a valgus knee; however, to obtain medial stability, only osteophytes from the medial femur and tibia were removed, while the primary medial static stabilizers, including the deep MCL, were preserved as much as possible [20].

After completing system setup, the extension and flexion gaps were adjusted using the dynamic balancing feature of the Mako system. The femoral component size was determined with reference to gap balancing. The medial extension gap was set to be equal to, or up to 1 mm wider than, the combined thickness of the femoral and tibial components. The lateral extension gap was then measured. In cases of persistent lateral tightness, the ITB attachment was partially released at the Gerdy’s tubercle, up to one-third of its width, and additional pie-crusting of the ITB was performed when necessary. If these procedures were insufficient to achieve optimal soft-tissue balancing, further a step-by-step lateral release was performed [21]. Patellar tracking was evaluated at this stage (see “Patellar tracking evaluation” section for details). The rotational alignment of the femoral component was then adjusted to optimize balancing, and the final extension and flexion gaps were determined.

### Bone resection procedure

**Fig. 3:**
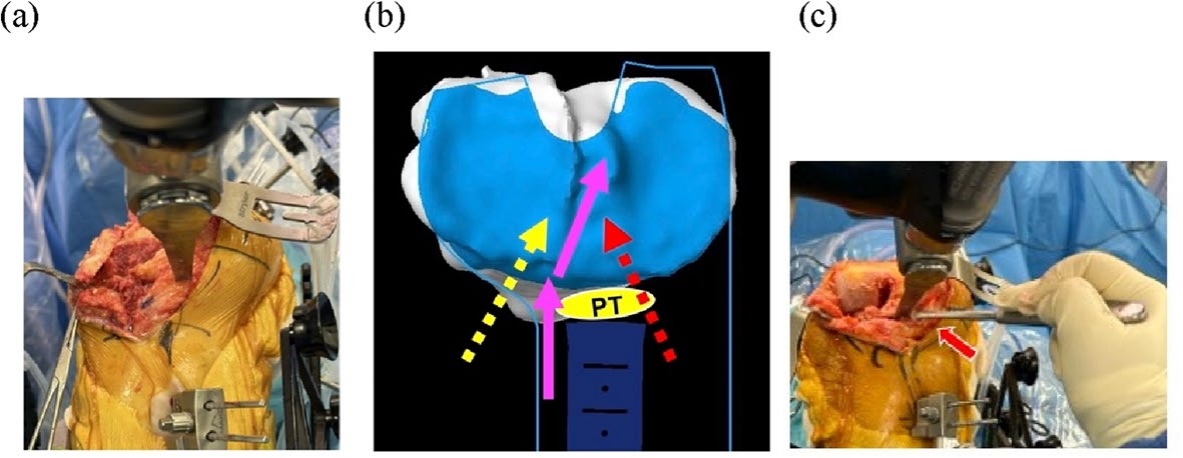
Proximal tibial bone resection. (**a**) Operative findings: As the patella was displaced medially, the robotic-arm-assisted bone blade moved directly toward the patellar tendon. (**b**) Proximal tibial surface in the Mako system: The program initially set the boundary (blue straight line) and blade trajectory through the patellar tendon (red dotted arrow). The manually intended lateral approach direction (yellow dotted arrow) was outside this programmed boundary, preventing robotic arm movement. Accordingly, the patellar tendon was protected using a surgical elevator. Bone resection was carefully performed between the tendon and the system boundary in the direction indicated by the pink solid arrow. (**c**) Operative findings: The patella was protected with a surgical elevator, and the proximal tibia was resected. PT: patellar tendon

Bone resections were performed using the robotic-arm-assisted bone blade. As the Mako program was originally designed for the medial approach, the movement of the robotic bone blade was directed toward the patellar tendon in the tibial resection (Fig. 3a). To prevent any damage, the patellar tendon was carefully protected with a surgical elevator, and bone resection was performed with particular caution while maneuvering between the patellar tendon and its adjacent boundary (Fig. 3b&c). After bone resection of the femur and tibia, the tibial implant size was decided, and the keel was created. In conventional TKA, soft-tissue balancing is evaluated and adjusted at this time. As soft-tissue balancing after bone resection was calculated and precisely achieved, additional soft-tissue release was not required in RTKA.

When a bone defect involved more than one-third of the surface area of the lateral compartment and exceeded a depth of 5 mm, additional bone resection was performed using the Mako system, followed by metal block augmentation [19].

### Onlay oval patellar implant

**Fig. 4:**
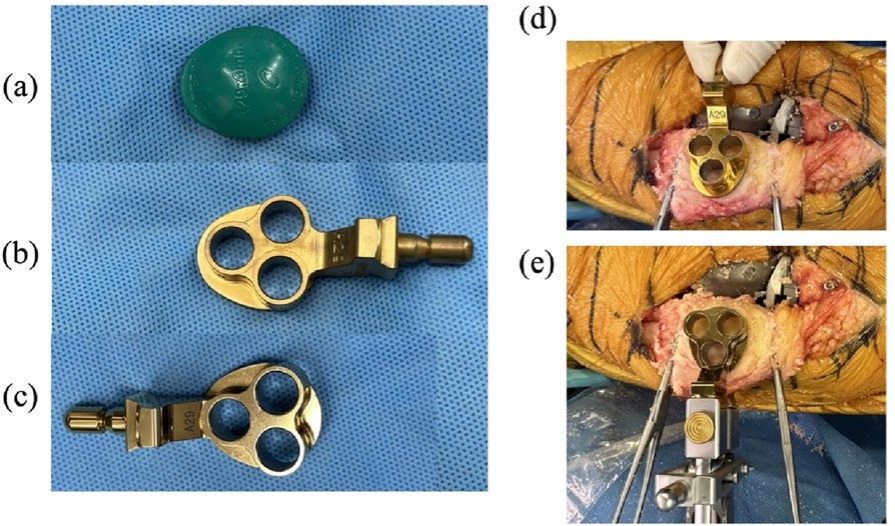
Onlay oval patella trial and drill templates. (**a**) Onlay oval patella trial. (**b**) Existing standard asymmetric patella drill template for the medial approach. (**c**) Originally designed a reversed-asymmetric patella drill template for the lateral approach. (**d**) The standard template could not be used for the lateral approach. (**e**) Our original device was able to be used for the oval implant during the lateral approach

For patellar resurfacing, three peg holes were created using a peg drill with our originally designed reversed-asymmetric patella drill template, specifically developed for the onlay oval implant in the lateral approach (Fig. 4 a–e). This patella drill template was created by Stryker and received approval in September 2024 from the medical device marketing authority of Japan.

### Patellar tracking evaluation

**Fig. 5:**
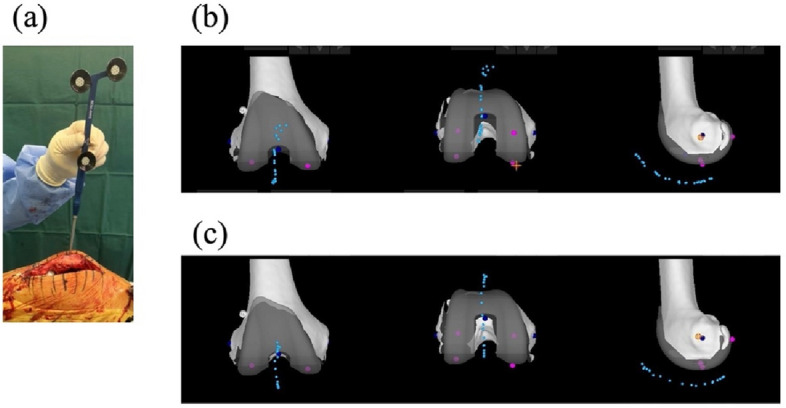
Evaluation of patellar tracking. (**a**) A 15-mm-deep hole was created, and the Mako knee sharp probe was inserted into this hole. The probe tip positions were recorded from full extension to maximal flexion to assess patellar tracking before bone resection (**b**) and after implantation (**c**). The patella was confirmed to track appropriately along the patellar groove after the lateral approach, before bone resection, and after implantation with three stitches of the lateral retinaculum

To evaluate the patellar tracking, a single hole approximately 15 mm deep (less than the patellar thickness) was crea*t*ed using a 2.0 mm in diameter Kirschner wire positioned at the center between the superior and inferior poles of the patella, slightly medial toward the articular surface of the patellar crest, without penetrating the articular cartilage. A Mako knee sharp probe, traceable by the robotic system, was inserted into the hole, and the probe tip position was recorded from full extension to maximal flexion to visualize the patellar tracking on the monitor (Fig. 5a). This assessment was performed in the recent three cases. Patellar tracking was examined twice: once after Mako system setup (before bone resection) and once after prosthesis implantation with three stitches in the lateral retinaculum (Fig. 5b&c).

Post-operative rehabilitation began on the first day after TKA, with patients allowed full-weight bearing as tolerated.

### Clinical outcome evaluation

Patients who underwent RTKA using the lateral approach provided written informed consent. This study assessed the clinical and radiographic outcomes of our RTKA procedures with modified utilization of the robotic technology, which were approved by our Institutional Review Board (Approval No. B-2020-355).

Changes in knee ROM measured on the examination table, Visual Analog Scale (VAS) scores, and Knee Injury and Osteoarthritis Outcome Score (KOOS) were evaluated pre-operatively and at the final follow-up. Negative knee extension values were recorded to indicate extension limitation.

Patients were examined at our outpatient clinic at 1, 2, 3, 6, and 12 months post-operatively and annually thereafter. Surgical complications, including peri-prosthetic joint infection, peri-prosthetic fracture, revision surgery, robotic pin-site complications, patellar complications, and readmissions related to RTKA within 90 days post-operatively, were assessed at these times.

### Radiological outcome evaluation

Anteroposterior and lateral knee radiographs, as well as full-length standing anteroposterior radiographs of the lower extremity, were obtained pre-operatively and post-operatively. Changes in the lateral femorotibial angle (FTA) and hip–knee–ankle (HKA) angle were evaluated. Negative values were recorded to indicate varus knee alignment.

To evaluate implant positioning in the coronal and sagittal planes, the alpha (α: femoral coronal), beta (β: tibial coronal), gamma (γ: femoral sagittal), and delta (δ: tibial sagittal) angles were measured on anteroposterior and lateral knee radiographs [22]. Because the anterior flange of the femoral component was designed with a 7° anterior slope, the γ angle was calculated as the angle between the anterior flange and the anatomical axis of the femur, minus 7°.

### Variation of knee laxity in TKA

Knee laxity in extension and flexion was defined as the difference between the lateral and medial joint gaps measured in the restrictive positions. Joint gaps were assessed after removal of the osteophytes and cruciate ligament(s), before bone resection, and again after prosthesis implantation. Variations in knee laxity before and after TKA were then evaluated.

### Statistical analyses

All data are presented as the median [first to third quartiles]. Statistical analyses were performed using IBM SPSS Statistics, version 29.0.1.1 (IBM Corp., Armonk, NY, USA). As all data were nonparametric, the Wilcoxon signed-rank test was used to compare clinical and radiographic outcomes. The level of statistical significance was set at *p* < 0.05.

## Results

Nine patients (ten knees) were evaluated. Of the ten knees, three cases were classified as KL grade III; the remaining cases were grade IV. Three patients presented with moderate valgus deformity that could not be corrected by the varus stress test under general anesthesia.

One patient had bilateral KL 4 valgus knee OA with severe scoliotic deformity and post-left total hip arthroplasty (THA) status and underwent simultaneous bilateral TKAs. Of the remaining eight patients, there was one KL 2 varus knee, two KL 2 valgus deformities, and five KL4 valgus knee deformities on the contralateral knees. Moreover, four patients showed scoliotic deformity in the spine, two patients had received ipsilateral THA, one patient had received bilateral THAs, and one patient had received ipsilateral THA and KL 3 hip OA on the other side without pain. Patient demographics are summarized in Table 1. Each case was evaluated independently.

**Table 1:** Patient demographics

Characteristics	Value
Patients	9 (10 knees); males: 2 (2 knees), females: 7 (8 knees)
Age at operation (years)	76.0 [69.8–77.0]
Follow-up duration (months)	12.0 [5.3–16.8]
Height (cm)	156.5 [149.6–161.9]
Weight (kg)	60.0 [49.5–63.3]
BMI (kg/m^2^)	23.7 [22.0–25.0]
Primary diagnosis	Primary OA: 8; post-traumatic arthritis: 1; rheumatoid arthritis: 1
KL classification	Grade III: 3; grade IV: 7
Ranawat classification	Type II: 7, type III: 3
Tibial insert type	CR: 2; PS: 8

BMI: body mass index, KL: Kellgren–Lawrence, OA: osteoarthritis, CR: cruciate-retaining, PS: posterior-stabilized. Data were shown as median [first and third quartiles]

Of the ten knees, the anterior portion of the ITB from the Gerdy’s tubercle was released in five knees. Additional pie-crusting of the ITB was performed in one knee. However, no further soft-tissue release was required. One patient underwent lateral tibial metal block augmentation with a tibial extension stem. As mediolateral instability decreased with the standard implants, constrained implants were not applied in these cases.

**Fig. 6:**
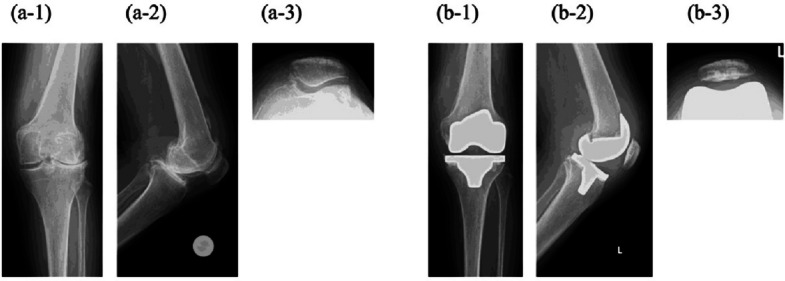
Radiographic findings in a 76-year-old woman. (**a**) pre-operative images: (1) anteroposterior, (2) lateral, and (3) skyline views. The KL classification was grade IV, the Ranawat classification was type II, FTA was 165°, and the HKA angle was valgus 11°. (**b**) post-operative image: (1) anteroposterior, (2) lateral, and (3) skyline views. The FTA and HKA angles improved to 175° and 0°, respectively. Implant positioning was accurate with an α angle of 96°, β angle of 89°, γ angle of 8°, and δ angle of 87°. The patellar position was appropriate on the skyline view. RTKA: robotic arm-assisted total knee arthroplasty, KL: Kellgren–Lawrence classification, FTA: lateral femorotibial angle, HKA: hip–knee–ankle angle

Following RTKA, the knee flexion angles decreased from 132.5° to 120.0°, and the knee extension angles significantly improved from − 5.0° to 0.0°. Moreover, VAS scores significantly decreased from 40.0 to 7.5, and all KOOS items significantly improved. Radiographic measurements of the FTA and HKA angle showed significant correction, and the accuracy of the femoral and tibial implant positioning was confirmed (Tables 2 and 3). Figure 6 shows a case of a 76-year-old woman with the Ranawat type II.

**Table 2:** Variation in clinical and radiographic outcomes

	**Pre-operative**	**Post-operative**	***p* value**
Extension (°)	− 5.0 [− 11.3 to − 3.8]	0.0 [0.0–0.0]	0.01
Flexion (°)	132.5 [117.5–135.0]	120.0 [118.8–125.0]	0.13
VAS	40.0 [29.3–55.0]	7.5 [0.0–13.5]	0.005
KOOS pain	39.0 [36.0–50.8]	75.0 [72.0–92.0]	0.012
Symptom	50.0 [36.8–70.3]	75.0 [72.0–86.0]	0.012
ADL	51.5 [37.0–62.0]	66.5 [44.0–83.3]	0.017
Sports/recreation	2.5 [0.0–23.8]	30.0 [13.8–43.8]	0.034
QOL	19.0 [7.8–28.0]	50.0 [31.3–59.8]	0.011
FTA	161.5 [145.3–166.3]	173.5 [169.8–176.0]	0.005
HKA angle	12.5 [6.8–27.0]	0.0 [− 1.0 to 0.8]	0.005

The knee flexion angle decreased, while the knee extension angle improved significantly. Moreover, VAS scores, all KOOS items, and radiographic evaluations showed significant improvement

VAS: Visual Analog Scale, KOOS: Knee Injury and Osteoarthritis Outcome Score, FTA: lateral femorotibial angle, HKA: hip–knee–ankle. Negative values indicate the limitation of knee extension and varus knee deformity. Data are shown as the median [first and third quartiles]

**Table 3:** Femoral and tibial implant positioning

Implant positioning	Value
Alpha angle (°)	95.8 [95.6–96.6]
Beta angle (°)	90.1 [89.4–90.3]
Gamma angle (°)	3.5 [2.5–4.7]
Delta angle (°)	88.3 [87.8–88.6]

Implant positioning was accurate using the mechanical alignment technique. Data are show*n* as the median [first and third quartiles]

The dynamic joint balancing technology was used to verify appropriate joint laxity in both extension and flexion (Table 4). Patellar tracking along the trochlear groove was clearly visualized and was found to be appropriate (Fig. 5b&c). There was no post-operative complication at the patellar holes.

**Table 4:** Variation of knee laxity

	Before resection	After implantation	*p* value
Extension	− 1.0 [− 3.3 to 0.63]	− 0.5 [− 1.3 to 1.0]	0.372
Flexion	0.0 [− 2.0 to 1.1]	0.75 [− 0.1 to 1.3]	0.046

Knee laxity before bone resection and after prosthesis implantation in extension and flexion. Knee laxity was defined as the difference between the lateral and medial gaps in each position. Before bone resection and after removal of the osteophytes and cruciate ligament(s), the medial gap was wider laterally, and the flexion gap was tighter. After implantation, the medial–lateral difference in extension diminished, and the lateral gap became slightly wider than the medial gap in flexion

Patients were observed for two to three weeks at the hospital and then once a month at the outpatient clinic. No case of peri-prosthetic joint infection, revision TKA, or RTKA-related readmission was observed in these cases.

## Discussion

The lateral approach combined with robotic technology was applied for TKA in patients with valgus knee deformity, achieving accurate implant positioning and adequate soft-tissue balancing. Appropriate patellar tracking was also confirmed with this surgical procedure and the use of onlay oval patellar implants.

The lateral approach is often used for severe valgus knees because it offers several advantages for arthrotomy and soft-tissue release [9]. However, the benefits of the lateral approach for valgus knees remain controversial [23, 24]. Although a step-by-step soft-tissue release has been reported, detailed procedures of soft-tissue release have not been as well established as those for the medial approach in TKA [21, 25]. Given that robotic technology has been shown to enable precise bone resection and optimal soft-tissue balancing, we applied it to valgus knee deformities treated through the lateral approach. Because the Mako system was originally programmed for use with the medial approach in primary TKA, there was an inherent possibility of patellar tendon damage when applying it for the lateral approach. Thus, careful control of the semi-active robotic arm, combined with the use of a surgical elevator to protect the patellar tendon, was essential for the procedure. With this modification, the patellar tendon was protected, and RTKA with the lateral approach could be performed as smoothly as with the medial approach. In cases with bone defects requiring metal block augmentation, additional bone resections were also performed accurately using the Mako system, similar to procedures performed via the medial approach [19].

Patellar resurfacing may be avoided in the presence of significant patellar thinning, which increases the risk of post-operative patellar fracture [26]. Conversely, registry-based evidence indicates that resurfaced cases demonstrate improved implant survivorship and reduced anterior knee pain compared with non-resurfaced cases [14]. Furthermore, PS implant designs have been associated with patellar clunk syndrome, for which resurfacing may offer benefit [27]. Based on these considerations, onlay oval patellar implants were used in our institution for all PS cases and for CR cases with moderate to severe patellar deformity. However, the conventional patella drill templates are primarily designed for the medial approach and are unsuitable for the lateral approach. Therefore, we designed a specialized onlay oval implant guide specifically for the lateral approach in TKA. This original device enables consistent and accurate implant placement while maintaining correct anatomical orientation and minimizing surgical error.

A surgical technique for RTKA in valgus knee using the lateral approach and functional knee positioning has been previously described [28]. Conversely, our approach employed mechanical alignment and the use of onlay oval patellar implants, which demonstrated favorable short-term clinical outcomes, optimal soft-tissue balancing, and satisfactory patellar tracking.

Several personalized alignment techniques have recently been proposed to restore prearthritic alignment rather than relying solely on the traditional mechanical alignment approach [29]. However, no consensus has yet been reached regarding the optimal alignment strategy in TKA [30]. Personalized alignment tends to facilitate the creation of mediolateral equal gaps with minimal soft-tissue release, particularly in varus knees. Nevertheless, physiologic knee gaps are inherently trapezoidal and asymmetrical (wider laterally than medially and wider in flexion than in extension [31]); therefore, the definition of an “optimal” gap remains debatable. In valgus knees, the medial gap is typically wider than the lateral gap. In our case series, medial stability was preserved as much as possible, while the lateral gap was increased through the lateral approach and selective soft tissue release. Consequently, the final gap was close to the rectangular gap compared with the pre-operative gap in valgus knees.

As the sample size was small, the follow-up period was short, and the surgeries were performed in a single institute, observations will continue with a larger cohort and longer follow-up in a multicenter study. Nevertheless, no cases of prosthesis malpositioning, limb malalignment, or malrotation (known causes of early TKA failure) were observed [32]. Patellar maltracking is another major concern associated with anterior knee pain and patellar complications; however, in our procedures, appropriate patellar tracking was achieved using the lateral approach with the onlay oval implant [33]. These findings suggest the potential for favorable long-term outcomes and implant longevity.

The main disadvantages of robotic technology are its high cost and the longer operative time compared with conventional TKA. However, it provides highly accurate limb alignment, optimal soft-tissue balancing, and precise patellar tracking, all of which can be visualized intra-operatively. Therefore, this technology holds promise for improving patient satisfaction and reducing the risk of early failure in both varus and valgus knee deformities.

## Conclusions

TKA for valgus knee deformity remains a challenging procedure. To address this, we applied the lateral approach, robotic technology, and the onlay oval patellar implant using our originally developed drilling device. The clinical and radiographic outcomes suggest that our procedures can facilitate more precise bone resection, optimal soft-tissue balancing, and proper patellar tracking for TKA in the case of valgus knee deformity.

## Data Availability

Aggregate data are presented in the Results section. The raw data, including individual ratings, are available from the corresponding author upon reasonable request.

## Abbreviations

CR: Cruciate-retaining
CT: Computed tomography
FTA: Femorotibial angle
HKA: Hip–knee–ankle
ITB: Iliotibial band
KL: Kellgren–Lawrence
KOOS: Knee Injury and Osteoarthritis Outcome Score
MCL: Medial collateral ligament
OA: Osteoarthritis
PS: Posterior-stabilized
ROM: Range of motion
RTKA: Robotic arm-assisted total knee arthroplasty
TKA: Total knee arthroplasty
THA: Total hip arthroplasty
VAS: Visual Analog Acale
